# Critical appraisal of the prognostic value of relative visceral fat area in localized clear cell renal cell carcinoma

**DOI:** 10.1097/MS9.0000000000003948

**Published:** 2025-09-23

**Authors:** Haseeb Safdar Ali, Huzaifa Noor, Esha Bilal, Muddassir Khalid, Supoma Ghosh Ria

**Affiliations:** a Department of Medicine, Nishtar Medical University, Multan, Pakistan; b Mugda Medical College, University of Dhaka, Bangladesh

*Dear Editor,*

I read with great interest the recent article by Feng *et al* on the prognostic value of relative visceral fat area (rVFA) in localized clear cell renal cell carcinoma (RCC)^[1]^. The paper studied the correlation between rVFA and recurrence-free survival in localized clear cell RCC and found it to be U-shaped and independently predictive of worse survival when high rVFA occurred. Although we appreciate the innovative application of CT-based fat quantification and machine-learning models, several methodological and interpretive details would be beneficial to clarify, which should strengthen the soundness, reproducibility, and practical relevance of the work of the authors. First, according to the recent guidelines on statistical reporting, which suggest either median [interquartile range (IQR)] or mean SD following data distribution^[2]^, Table 1 incorrectly labels age as mean SD but presents median (IQR). Such a discrepancy can mislead readers and needs to be fixed. Similarly, since epidemiologic norms emphasize the necessity to identify non-biologically feasible values such as extreme body mass index (BMI) outliers^[3]^, the specified BMI SD of 21.74 at Quartile 3 seems extraordinarily doubtful and will have to be confirmed. Second, the hazard ratio of 62.05 with the low limit of the CI (62.05, 825.75) is statistically inaccurate and represents either overfitting of the model or few events; the precision around the limit would usually differ^[4]^. Besides, visceral to subcutaneous fat ratio is a well-established prognostic factor in oncology^[5]^, and omitting subcutaneous fat area despite its insignificant *P* value in univariate analysis (*P* = 0.050), which is borderline inadequate, and its direct effect on rVFA without sufficient explication makes results difficult to discern.

Finally, it is necessary to speak about several editorial problems. There is no update in the ethics approval section, as it contains the name of the ethics-approved authority as XXX rather than the name of the institute. Further, there are misattributed references: in particular, Reference 24, which cites leptin-related mechanisms in RCC, discusses the loss of the Y chromosome. Although they are trivial, these mistakes affect the integrity and finesse of what otherwise would be a significant piece of writing. To summarize, we applaud the efforts of Feng *et al* on the prognostic value of CT-based fat quantification in clear cell RCC, as this aspect is of apparent clinical significance. Their study provides a valuable perspective on how body composition could factor into oncologic outcomes. We are convinced that improving the methodological and interpretive aspects of the points we mentioned will make this study more robust and provide a more solid foundation for future research, ultimately contributing to risk stratification and patient care.

TITAN Guidelines: This manuscript complies with TITAN Guidelines, 2025, declaring no use of artificial intelligence^[6]^.

## Data Availability

Not applicable.

## Research registration unique identifying number (UIN)

Not applicable for this letter to the editor.
